# Primary Central Nervous System T-cell Lymphoma Associated With Hepatitis B and D Virus Coinfection

**DOI:** 10.7759/cureus.14394

**Published:** 2021-04-09

**Authors:** Saqib R Khan, Saad Nasir, Muhammad Tariq, Zoya A Siddiqui, Munira Moosajee

**Affiliations:** 1 Medical Oncology, Aga Khan University Hospital, Karachi, PAK; 2 Internal Medicine, Aga Khan University Hospital, Karachi, PAK

**Keywords:** hepatitis d, t-cell lymphoma, hepatitis b virus

## Abstract

Primary central nervous system lymphoma (PCNSL) is a rare type of non-Hodgkin’s lymphoma. It is defined as lymphoma of the central nervous system without any systemic disease elsewhere at the time of diagnosis. Based on the phenotypical features, it is divided into two categories, B-cell and T-cell, with the latter being less common. Viruses, such as human immunodeficiency virus (HIV) and human T-lymphotropic virus type 1 (HTLV-1), are linked to the T-cell variant; however, there is a lack of evidence suggesting associating hepatitis B and D virus coinfection with it. We report a case of a 34-year-old male who presented with T-cell PCNSL and was later diagnosed with a hepatitis B and D virus coinfection.

## Introduction

Primary central nervous system lymphomas (PCNSLs) are defined as lymphoma of the central nervous system (CNS) without a primary tumor elsewhere. PCNSL is rare, with the most common subtype being diffuse large B-cell lymphoma. The incidence of PCNSL is approximately 30 cases per million person-years [1]. B-cell PCNSL makes up the majority of cases, with primary central nervous system T-cell lymphoma (PCNSLT) being extremely uncommon. Studies report an incidence of approximately 2%-8% of PCNSLT among all patients with PCNSL [2]. Choi et al. in their study described a higher percentage of T-cell lymphomas (16.7%, 7/42 cases) in their series of primary CNS lymphomas from Korea [3]. Viral infections have been implicated in contributing to the pathogenesis of PCNSL. There is a lack of evidence supporting the association of PCNSLT with hepatitis B and D coinfection. Here, we report a case of a 34-year-old male with hepatitis B and hepatitis D virus coinfection, who was later diagnosed with PCNSLT after taking verbal consent.

## Case presentation

A 34-year-old male presented to the emergency department with generalized tonic-clonic seizures and altered mental sensorium. His Glasgow coma scale (GCS) was 3/15, and he was immediately intubated and later shifted to the intensive care unit (ICU). His family members had noticed mood fluctuation, irritability, and difficulty in concentrating for the past one week accompanied by low-grade recurrent fever at home not associated with rigors or chills. He was also found to have a loss of appetite and episodic headaches for the last two months.

**Figure 1:**
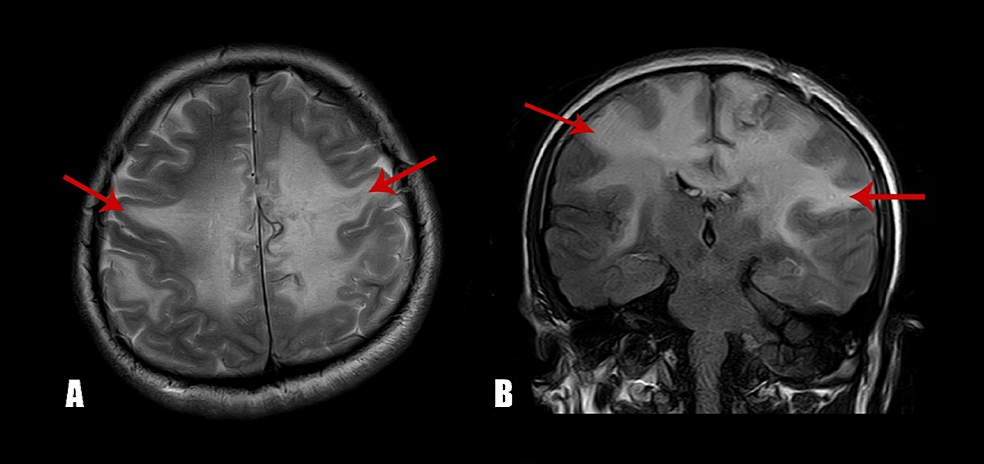
Magnetic resonance imaging of the brain at the time of diagnosis. (A) Axial, T2 section showing abnormal signaling in bilateral white matter consistent with primary disease. (B) Coronal view showing extensive areas of T2/FLAIR hyperintense signal abnormality identified in the supratentorial region involving bilateral cerebral hemispheres. FLAIR: Fluid-attenuated inversion recovery

**Figure 2:**
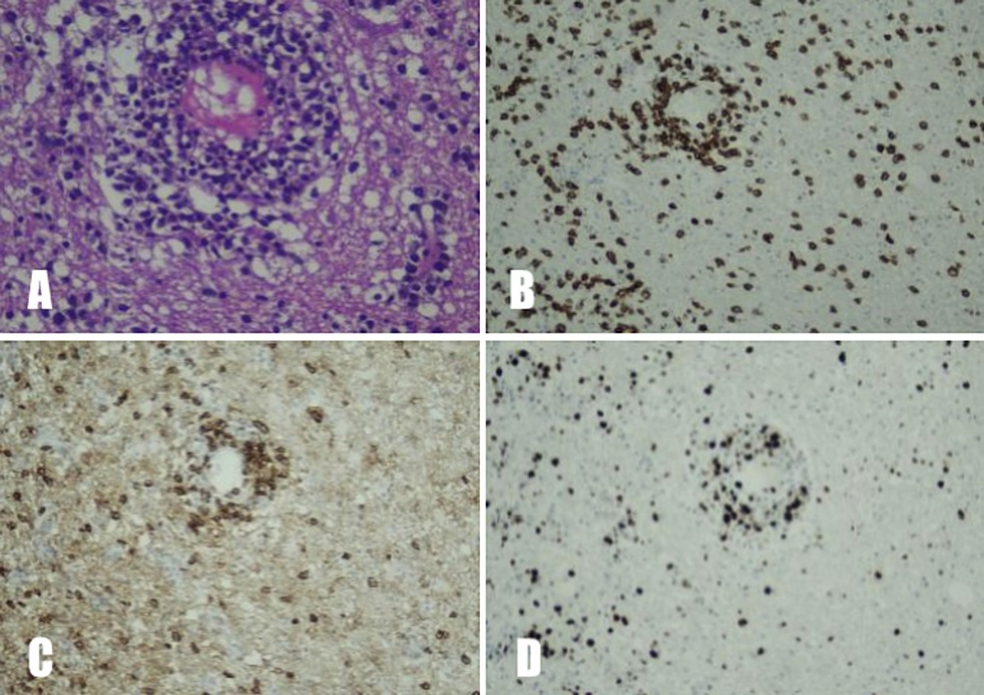
(A) H&E stain of brain parenchyma showing an atypical lymphoid infiltrate with perivascular cuffing. (B) Predominant atypical lymphoid population showing positivity for CD3 immunostain. (C) CD4 immunostain highlighting the predominant atypical T-lymphoid population. (D) Ki-67 showing a high proliferative index in the atypical lymphoid population. CD: Cluster of differentiation H&E: Hematoxylin and eosin

On examination, he had an average built and his vitals were in the reference ranges. General physical examination was unremarkable except for pallor. There were no lymph nodes palpable. On central nervous examination, he was drowsy and not oriented to time, place, and person. Examination of other systems and fundoscopy were unremarkable. A computed tomography (CT) scan of the head was performed, which showed no infarct, intracranial hemorrhage, or mass effect. Magnetic resonance imaging (MRI) of the brain showed multiple, asymmetrical gyral abnormal signal intensities involving bilateral cingulate gyrus, bilateral frontoparietal lobes, and bilateral insular cortices with the mild enhancement of leptomeninges in the left parietal lobe (Figure 1A, 1B). He was started on broad-spectrum antibiotics for suspected meningitis; however, cerebrospinal fluid (CSF) BioFire FilmArray culture showed no growth of any organism, and CSF cytology was negative as well. His condition deteriorated over time and a repeat MRI brain with magnetic resonance angiography (MRA) was done after a week which showed symmetrical cortical and subcortical signal abnormality in bilateral cerebral and cerebellar hemispheres with interval progression as now the involvement of the corpus callosum as well and a possibility of gliomatosis cerebri. Magnetic resonance spectroscopy (MRS) revealed features were favoring neoplastic lesions. Systemic scans were negative for any disease. He then underwent a left frontoparietal lobe biopsy. Histopathology was suggestive of a T-cell lymphoproliferative disorder with atypical lymphoid cells (Figure 2A) predominantly showing positivity for CD3 and CD4 immunohistochemical stains (Figure 2B, 2C). The Ki-67 showed a proliferative index of approximately 50-60% (Figure 2D). CD20, CD30, terminal deoxynucleotidyl transferase (TdT), and anaplastic lymphoma kinase (ALK) were negative.

**Figure 3:**
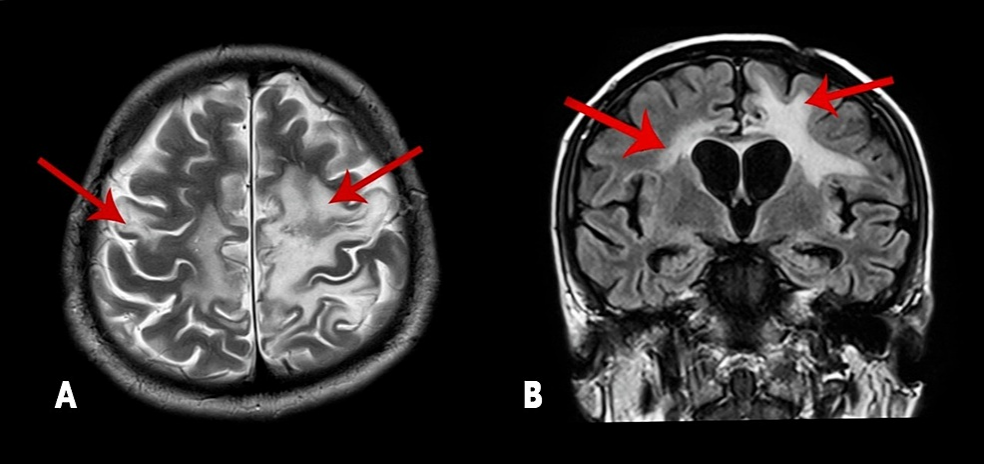
Magnetic resonance imaging of the brain after chemotherapy. A: Axial, T2 section showing significant disease reduction as seen by T2/FLAIR white matter signal abnormality within bilateral supratentorial cerebral hemispheres; B: Coronal view showing significant reduction in the disease process. FLAIR: Fluid-attenuated inversion recovery

His laboratory investigation was negative for human immunodeficiency virus (HIV) serology and hepatitis C infection; however, he was positive for hepatitis B and D virus antigens. He was subsequently started on entecavir. For management of PCNSLT, he was started on the modified DeAngelis protocol which comprises alternating cycles of MVP (Methotrexate 3.5 g/m^2^, Vincristine 1.4 g/m^2^, Procarbazine 100 mg/m^2^) with MV (Methotrexate 3.5 g/m^2^, Vincristine 1.4 g/m^2^). His neurological status improved significantly following the administration of chemotherapeutics. MRI brain was repeated after five cycles of MVP that showed a significant reduction in the disease. He then received an additional two cycles of MVP as per protocol. An autologous stem cell transplant as consolidation treatment was offered, but because of financial constraints, he received whole-brain radiation instead. On follow-up, an MRI brain was done, which showed a significant improvement in the disease process (Figure 3A, 3B). His condition continued to improve, and a few months later, he was able to resume his daily routine.

## Discussion

PCNSL is a rare variant of non-Hodgkin lymphomas. Most of these tumors are of B-cell phenotype with diffuse large B-cell lymphoma being the most common subtype. PCNSLT can appear at any age, with a median age of about 60 years at diagnosis, and has a slight male predominance [4]. Most affected patients are immunocompetent. However, there are some reported cases of PCNSLT in patients with HIV or human T-lymphotropic virus type 1 (HTLV-1) infections [4]. Epstein-Barr virus (EBV) plays a role in B-cell lymphomas, especially in immunocompromised patients, but the virus is not linked to PCNSLT [5]. A study reported the prevalence of HBV infection in PCNSL as 16.1% [6]. The association of viral hepatitis and PCNSLT has not been described in the literature. Our patient was negative for HIV. HTLV-1 was not checked because of the unavailability of the test at our institution. He was coincidentally found to be positive for hepatitis B and hepatitis D virus antigens.

As a spectrum of cells makes up the tumor milieu, therefore, making it difficult to differentiate T-cell lymphomas from reactive lesions. The diagnosis is further complicated because, in T-cell lymphomas, the immunophenotyping may not be definitive; as T-cells do not express clonal markers like B-cells do such as immunoglobulin light chains [7]. Staining by immunohistochemistry (IHC) is useful in determining the cell lineage in which these atypical T-cells are immunoreactive for CD3 and any combination of CD2, CD4, CD5, CD7, and/or CD8. Clonality can be determined by T-cell gene rearrangement, where the diagnosis is still in question [7]. In our patient, the clinical picture, morphology, and IHC pattern were consistent with PCNSLT. To prioritize the finances of the patient, we did not perform the T-cell gene rearrangement.

Several prospective and retrospective trials have evaluated different treatment protocols for PCNSL [8]. High-dose methotrexate is the cornerstone in the management of PCNSL. Incorporating radiation and autologous stem cell transplant has resulted in an improvement in survival. Historically, the prognosis is poor, with a five-year disease-specific survival of 17% [4]. However, with the introduction of newer treatment modalities, this has improved, as a recent study reports overall survival of 65% at two years [9]. Survival analysis is primarily reported for B-cell PCNSL. Limited data are available for PCNSLT. One such case series from China reports the two- and five-year-disease-specific survival of 51% and 17%, respectively, which is similar to B-cell PCNSL [10].

Through this article, we aim to highlight a rare association of hepatitis B and D virus coinfection with PCNSLT. Since this is the first reported case, the pathophysiology involved is yet to be determined. Our case limitations include an inability to rule out simultaneous HTLV-1 infection because of the unavailability of testing at our facility. 

## Conclusions

PCNSLT is extremely rare brain tumors. There is a potential association between hepatitis B and D coinfection and PCNSLT, therefore all viral markers should be checked to provide adequate management. Imaging studies and CT-guided biopsy with immunohistochemistry should be performed for definitive diagnosis. As there is a lack of effective treatment options currently available for PCNSLT, we further recommend clinical trials, to develop an optimized treatment plan for these patients.
